# Shape-based separation of microalga *Euglena gracilis* using inertial microfluidics

**DOI:** 10.1038/s41598-017-10452-5

**Published:** 2017-09-07

**Authors:** Ming Li, Hector Enrique Muñoz, Keisuke Goda, Dino Di Carlo

**Affiliations:** 1 Department of Bioengineering, University of California, Los Angeles, USA; 2 Department of Electrical Engineering, University of California, Los Angeles, USA; 3 Department of Chemistry, University of Tokyo, Tokyo, Japan; 4 Japan Science and Technology Agency, Tokyo, Japan; 5 California NanoSystems Institute, University of California, Los Angeles, USA; 6 Jonsson Comprehensive Cancer Centre, University of California, Los Angeles, USA

## Abstract

*Euglena gracilis* (*E. gracilis*) has been proposed as one of the most attractive microalgae species for biodiesel and biomass production, which exhibits a number of shapes, such as spherical, spindle-shaped, and elongated. Shape is an important biomarker for *E. gracilis*, serving as an indicator of biological clock status, photosynthetic and respiratory capacity, cell-cycle phase, and environmental condition. The ability to prepare *E. gracilis* of uniform shape at high purities has significant implications for various applications in biological research and industrial processes. Here, we adopt a label-free, high-throughput, and continuous technique utilizing inertial microfluidics to separate *E. gracilis* by a key shape parameter-cell aspect ratio (AR). The microfluidic device consists of a straight rectangular microchannel, a gradually expanding region, and five outlets with fluidic resistors, allowing for inertial focusing and ordering, enhancement of the differences in cell lateral positions, and accurate separation, respectively. By making use of the shape-activated differences in lateral inertial focusing dynamic equilibrium positions, *E. gracilis* with different ARs ranging from 1 to 7 are directed to different outlets.

## Introduction

*Euglena gracilis* (*E. gracilis*) is a species of single-celled eukaryotic microalgae that can grow both autotrophically and heterotrophically on non-agricultural land. It has been attracting great industrial and academic interests for its ability to synthesize valuable products with commercial applications, such as paramylon, wax esters, and various types of nutrients (i.e. vitamins, minerals, fatty acids, and amino acids). *E. gracilis* accumulates paramylon, an insoluble β-1,3-glucan, under aerobic conditions, which has been shown to be able to stimulate immunomodulating activities^1^, treat and prevent infectious, oncologic and allergic diseases (i.e. human immunodeficiency virus-HIV^2^, colon cancer^3^, and atopic dermatitis^4^), and fabricate nanomaterials (i.e. nanofibers)^5^. Wax esters, mainly composed of C14:0 saturated fatty acid, myristic acid, and myristyl alcohol^6^, synthesized under anaerobic conditions, holds promise for biodiesel and jet fuel production as an alternative to fossil fuels. Moreover, *E. gracilis* has been reported to be capable of producing dietary supplements α-tocopherol and β-carotene^7^, as well as assay vitamin B12 in body fluids^8^, waste water quality^9^, and ecotoxicity of engineered nanoparticles^10^. Thus, microalgae such as *E. gracilis* may be able to impact a large number of fields, including renewable bioenergy, nutritional and functional food, environment, and health-care.

**Figure 1:**
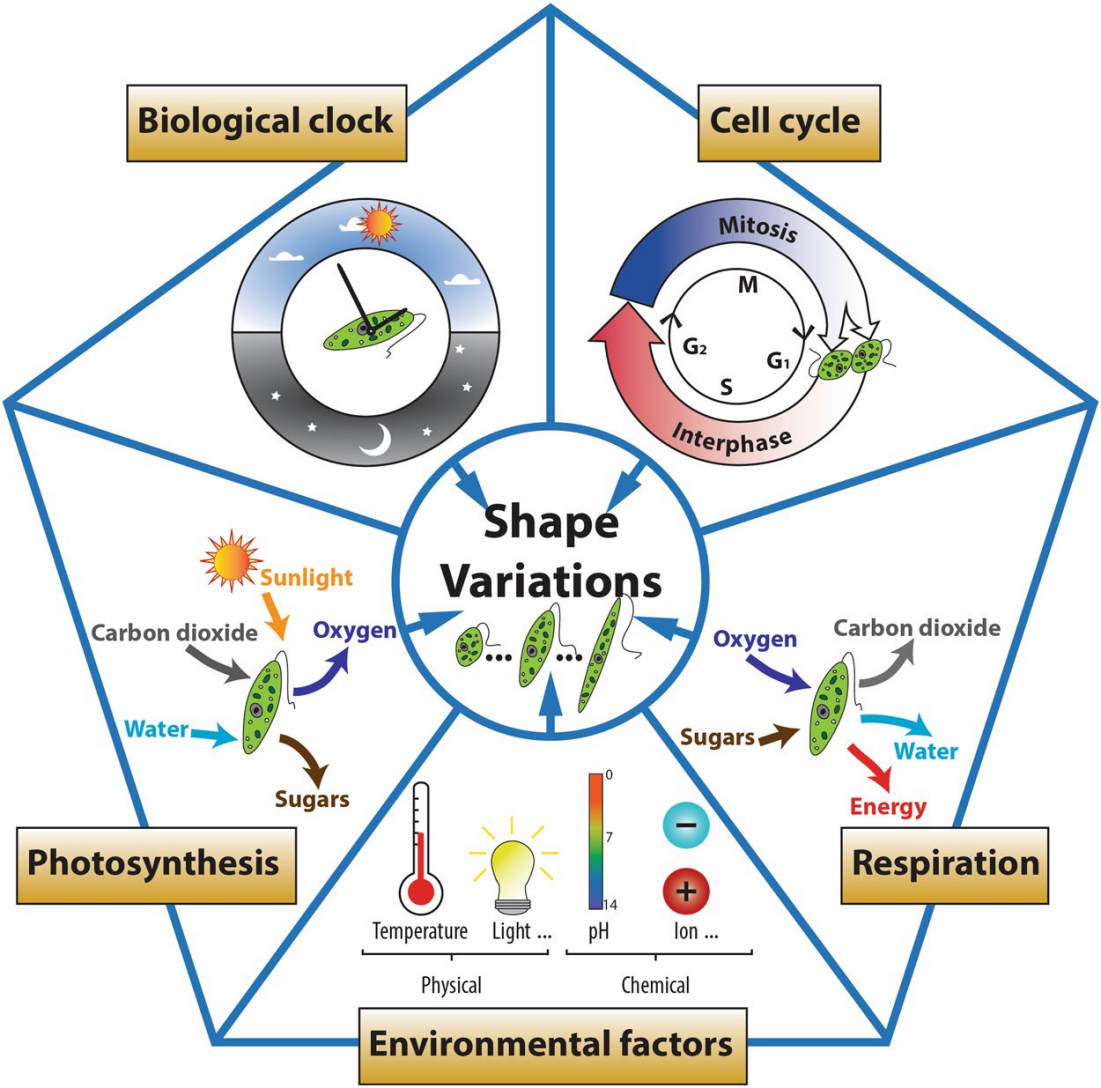
The variation of shape in *E. gracilis* is correlated to biological clock, photosynthesis, respiration, cell cycle, and environmental factors.

The variation in shape represents one of the most important noticeable characteristics for *E. gracilis*. The microalga can vary in shape dramatically, ranging from nearly spherical (aspect ratio, AR = 1) to elongated cylindrical (AR > 5) with most cells appearing spindle-shaped. The shape of *E. gracilis* has been demonstrated to be closely correlated to biological clock, photosynthesis, respiration, cell cycle, and environmental factors (Fig. 1). When grown under the synchronizing effect of a repetitive daily light-dark cycle, *E. gracilis* displays two shape changes per day^11^: the cell population increases in length up until the middle of the light cycle, exhibiting an elongated cylindrical shape, and decreases in length for the remainder of the 24-hour period, exhibiting a nearly spherical shape at the end of the dark cycle. These shape variations reflect a circadian rhythm, and have been demonstrated to be controlled by the biological clock^12^. Photosynthetic and respiratory reactions have also been reported to be involved in daily *E. gracilis* shape changes^11, 13^: photosynthetic electron transport pathways are necessary for the shape change from spherical to elongated, while respiratory pathways are involved in both the spherical to elongated and the elongated to spherical shape changes. Shape is also a cell cycle biomarker for *E. gracilis*, which reproduces by longitudinal binary fission, and a decrease in cell length could be a signal for the onset of mitosis. Moreover, the shape can vary in response to a variety of physical and chemical treatments, including light, temperature, pH, and cation concentration^14–17^.

In order to develop metabolically and genetically engineered *E. gracilis* with superior characteristics for low-cost biodiesel and biomass production, it would be important to prepare shape-synchronized *E. gracilis* cell populations adopting relatively uniform shape and size at high purities. The study of a homogeneous *E. gracilis* population allows for a better understanding of the biochemistry and molecular biology of valuable derivatives (i.e. paramylon and wax esters) production, along with nuclei and subcellular organelles (i.e. mitochondrion, chloroplasts, flagella, and pellicles). Shape-based separation of *E. gracilis* can provide an enriched cell population at a single stage, with the same status or phase. This would improve the investigation of correlated physiological and metabolic signatures by gene expression profiling. To date, sequencing the genome of *E. gracilis* has proved problematic due to its complexity^18^. More importantly, the shape-based separation, enrichment, and synchronization of *E. gracilis* open venues for a wide range of downstream applications, including biochemical assays, mutational analysis, and next generation sequencing.

However, shape has not been considered in traditional separation methods, such as pore-based filtration and centrifugation. Several microfluidic techniques have been utilized for shape-based separation of particles. Some techniques are based on internal channel or microstructure topology induced microflow, such as hydrodynamic filtration (HDF)^19^, and deterministic lateral displacement (DLD)^20, 21^. But these techniques require complex networks of microscale structures (i.e. branched channels and post arrays, respectively, for HDF and DLD), and require low flow rates (i.e. <10 µL/min). Other techniques integrate microfluidic flow with externally applied electrical^22^ or magnetic^23^ fields. However, these techniques require more device fabrication and external setup, along with the operation at relatively slow flow rates. More recently, viscoelastic fluids^24, 25^ have been demonstrated as another effective approach to sort spherical and peanut shaped microparticles due to the balance between elastic lift and inertial forces. Although this technique is high-throughput and simple-to-integrate, a precise control over the rheological properties of suspending medium is needed, limiting its practical applications.

Here, we utilize inertial focusing techniques^26, 27^ to separate *E. gracilis* by shape in a label-free, high-throughput, and cost-effective manner. Under the combined effects of two inertial lift forces (i.e. a shear-gradient lift force and a wall-effect lift force), particles migrate to dynamic equilibrium positions across fluid streamlines. Inertial microfluidics has been extensively studied to focus, order, and separate spherical particles, and mammalian cells with relatively constant size and shape, yet there have been few reports about inertial effects on shaped biological and synthetic particles. Hur *et al*.^28^ characterized the inertial effects on polymeric particles with various shapes, such as cylindrical, doublet-shaped, and disk-shaped. Masaeli *et al*.^29^ demonstrated the ability of inertial microfluidics to separate polystyrene beads and yeast cells based on shape. We recently showed that inertial focusing can be utilized together with secondary flows to focus *E. gracilis* cells varying in shape to a single focal stream^30^. However, it has not been shown whether inertial lift forces can be used to separate microalgae based on shape. In this work, we investigate the behaviors of *E. gracilis* cells with different ARs ranging from 1 to 7 under inertial effects in a straight microchannel region, an expansion region, and outlets. As the dynamic inertial focusing equilibrium positions are dependent on *E. gracilis* cell AR, *E. gracilis* with particular ARs migrate to distinct lateral equilibrium positions, and exit from different outlets. To the best of our knowledge, this is the first time that separation and enrichment of a single species of microalgal cells by shape has been reported using microfluidics.

## Results

**Figure 2:**
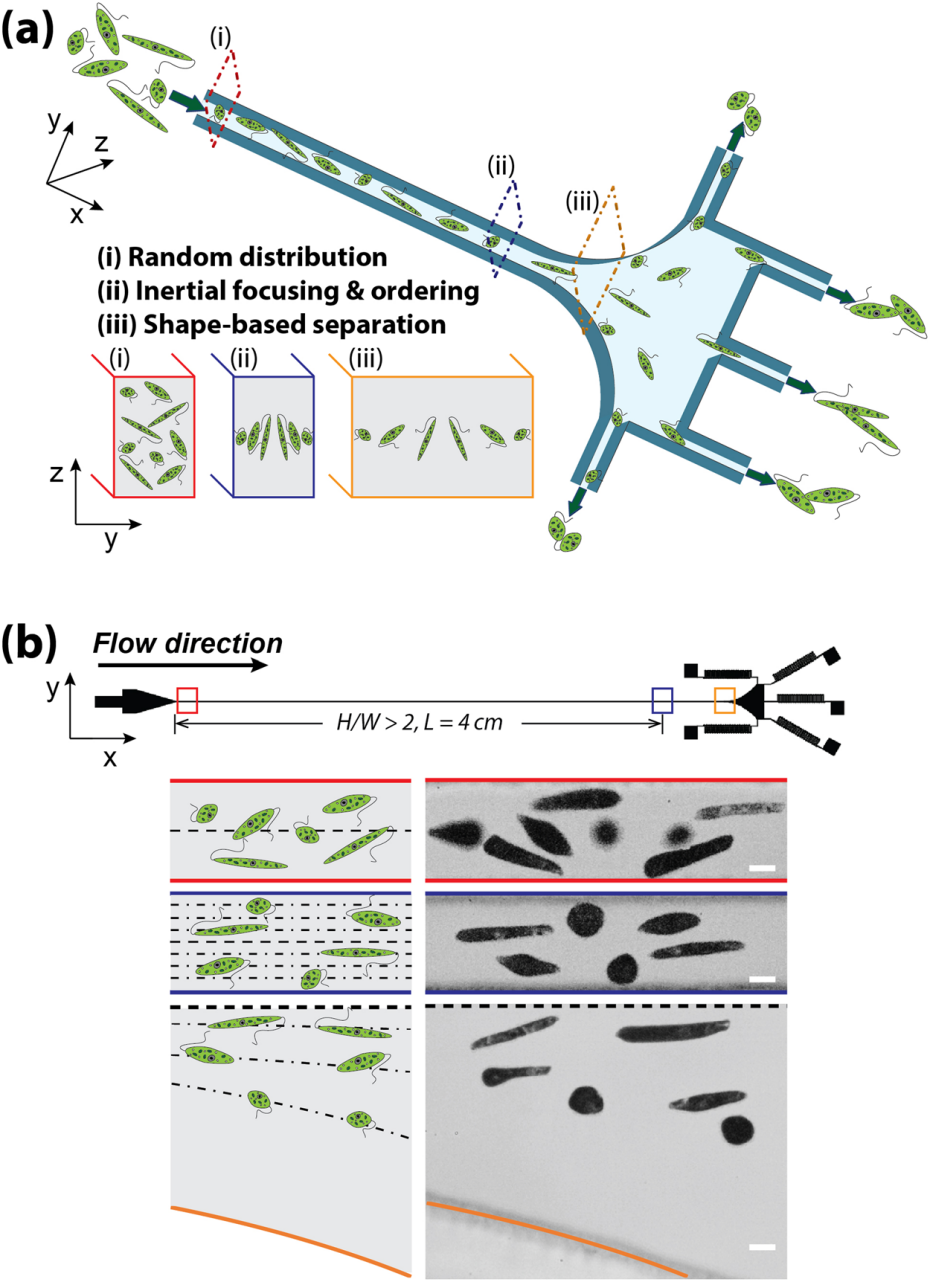
Shape-based separation of *E. gracilis* with different aspect ratios due to shape-dependent lateral inertial focusing equilibrium positions. (**a**) A schematic view of the inertial microfluidic channel and separation principles. (**b**) A top view (upper) showing the structure and dimensions of the microfluidic device which consists of an inlet, a straight rectangular microchannel, an expansion region, and five outlets with fluidic resistors. Schematics (lower left) and superimposed experimental images (lower right) illustrating the distribution of *E. gracilis* cells with different ARs at the inlet, downstream, and expansion region. Scale bar = 10 µm.

The microfluidic device used in the experiment consists of an inlet, a high aspect ratio (height/width >2) straight microchannel, a gradually expanding region, and five branched outlets each terminated with fluidic resistors, i.e. serpentine channels (see Fig. 2). The Reynolds number, Re = *ρνD*_*h*_ */μ*, is a dimensionless parameter describing the ratio between inertial and viscous forces^26^. Here, *ρ*, *ν*, and *μ* are the density, mean velocity, and dynamic viscosity of the fluid, respectively. *D*_*h*_ = *2WH/W+H* is the hydraulic diameter of the channel, where *W* and *H* are the channel width and height, respectively. At finite Re, the movement and position of target *E. gracilis* cells in the three regions indicated by sequence numbers i, ii, and iii, are distinct: (i) *E. gracilis* cells are randomly distributed close to the inlet; (ii) *E. gracilis* cells with various shapes generally migrate to two equilibrium positions along the long faces of the microchannel due to the balance between a shear-gradient lift force and a wall-effect lift force, and long rod-shaped cells migrate to positions closer to the channel centerline than spherical ones; (iii) the differences in shape-dependent lateral equilibrium positions are enhanced while cells continue along their focusing streamlines (Fig. 2a). Finally, *E. gracilis* cells with variable shapes are directed to different outlets: spherical cells exit from the outlets closest to the channel sidewalls, while longer rod-shaped cells are collected from the outlet closer to the channel centerline. Figure 2b presents schematically and experimentally the *E. gracilis* cells with various ARs at the inlet, 4-cm downstream of the inlet, and expansion region.

### Lateral equilibrium positions depend on *E. gracilis* cell aspect ratio

**Figure 3:**
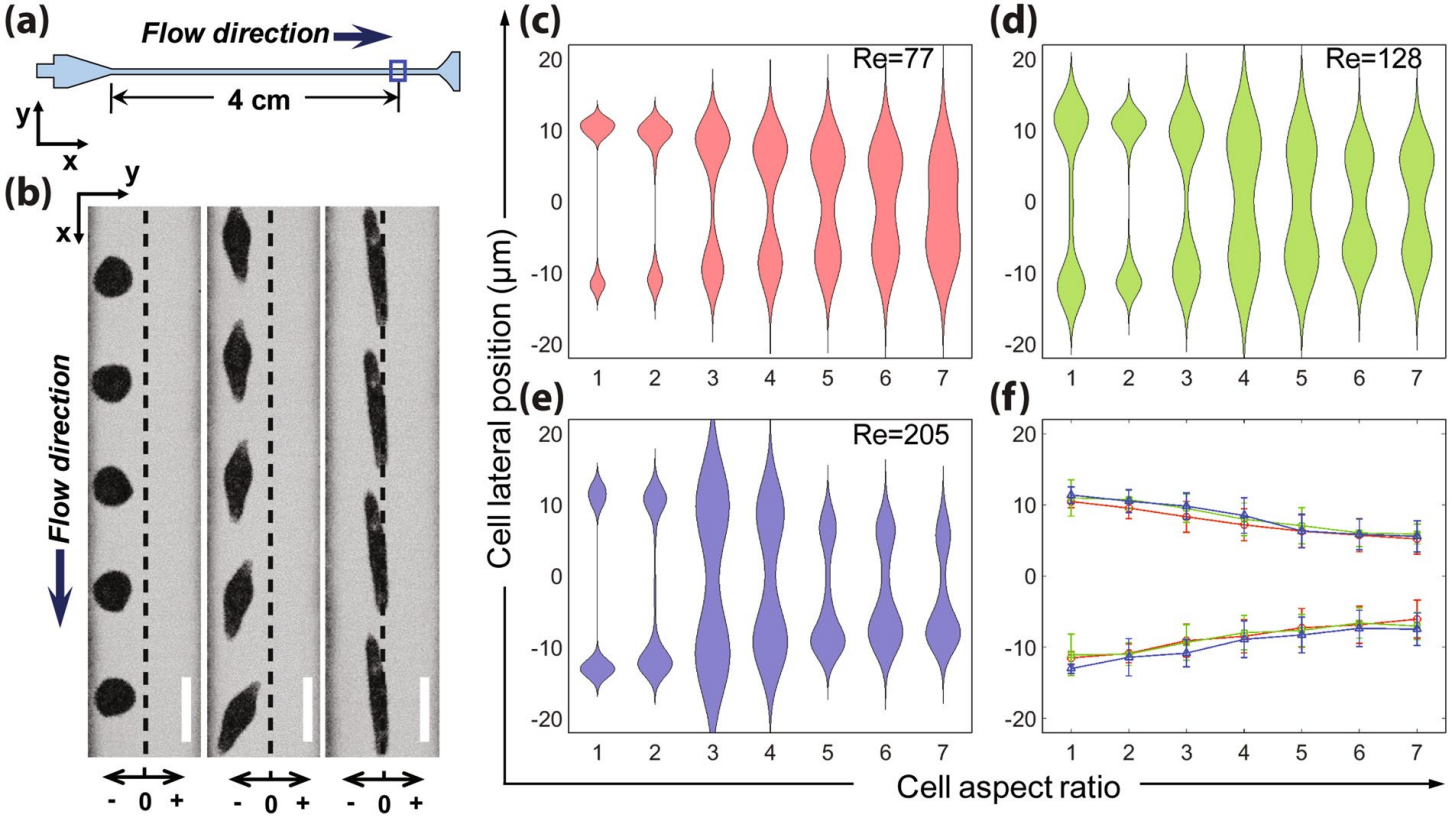
Lateral (Y-) focusing distributions depend on *E. gracilis* cell aspect ratio (AR). (**a**) An illustration of a straight rectangular channel region used for measuring the lateral positions of *E. gracilis* flowing through the microfluidic channel. (**b**) Superimposed experimental images showing the lateral inertial focusing equilibrium positions for *E. gracilis* with different ARs at Re = 77. Scale bar = 20 µm. (**c**–**e**) Violin plots of lateral positions for *E. gracilis* cells with ARs ranging from 1 to 7 for Re = 77, 128 and 205, respectively. (**f**) Averaged equilibrium positions are plotted as a function of cell AR for three Re. The error bars indicate the standard deviations for at least 100 measurements.

*E. gracilis* cells with different ARs have different lateral (Y-) inertial focusing equilibrium positions. The effect of cell AR on lateral equilibrium positions is investigated by flowing *E. gracilis* cells with different ARs ranging from 1 to 7 at three flow rates, 300, 500, and 800 µL/min. The corresponding Re are 77, 128 and 205, respectively. We chose seven levels of AR with a spacing of 1 in order to give readers a higher definition view of the dynamics and lateral equilibrium position distributions for *E. gracilis* cells having different shapes. We chose a straight rectangular channel region, 4-cm downstream of the inlet (Fig. 3a), for the measurement in order to ensure effective inertial focusing and ordering effects. The violin plots visualize the distribution of lateral focusing distributions for *E. gracilis* with different ARs, and its probability density at different values. In all three flow conditions, there are two sets of lateral equilibrium positions symmetric about the channel centerline for *E. gracilis* cells with all ARs (Fig. 3c–e). However, spherical *E. gracilis* cells migrate to locations closer to the walls compared to cells that have higher AR. In contrast, long rod-shaped cells occupy equilibrium positions much closer to the channel centerline than cells that have lower AR. The averaged lateral positions demonstrate that the distance to the channel centerline decreases with increasing AR (Fig. 3f). Similar AR-dependent inertial focusing behaviors were observed in a 50-µm-wide, 90-µm-high microchannel (see Supplementary Fig. S1). These results agree with previous numerical analysis by Gossett *et al*.^31^ and experimental studies by Masaeli *et al*.^29^: there are usually two preferred equilibrium positions along the longer channel faces in a rectangular microchannel, and rod-like microparticles migrate to a stable position closer to the channel centerline than that of spherical ones with the same volume. With finite inertia, ellipsoid *E. gracilis* cells undergo rotational motion in the microchannel. Compared to lower AR *E. gracilis* cells, higher AR ones possess inertial focusing equilibrium positions further from the channel wall due to their larger rotational diameter, resulting in larger wall-effect lift force in the near-wall region, which is much stronger than the balancing shear-gradient force, and directs the cells further away from the wall^29^.

### Expansion region enhances the differences in lateral equilibrium positions

**Figure 4:**
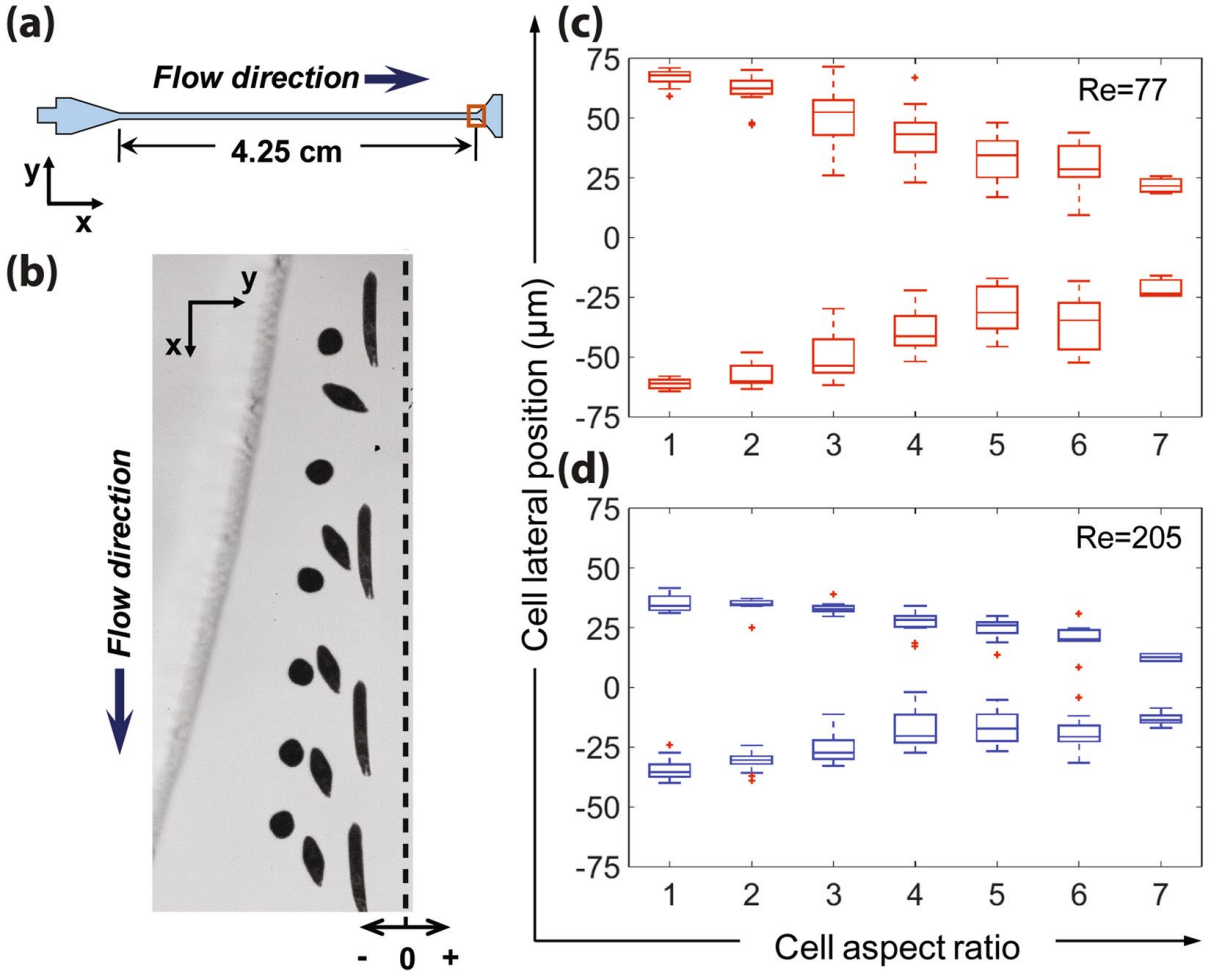
The gradually expanding region enhances the differences in AR-dependent lateral positions while maintaining *E. gracilis* cells in the focusing streamlines. (**a**) An illustration of an expansion region used for measuring the lateral positions of *E. gracilis* flowing through the microfluidic channel. (**b**) A superimposed experimental image showing the lateral positions for *E. gracilis* with different ARs at Re = 77 (half of the expansion region is shown). Scale bar = 20 µm. (**c**,**d**) Spread plots and box plots of lateral positions for *E. gracilis* cells with different ARs ranging from 1 to 7 for Re = 77 and 205, respectively.

A gradually expanding region is designed downstream of the straight rectangular microchannel to enhance the differences in AR-dependent lateral inertial focusing equilibrium positions, enabling high-purity shape-based separation of *E. gracilis* cells at the outlets. *E. gracilis* cells with different ARs are inertially focused to two lateral equilibrium positions after flowing through the straight microchannel, while the expansion region gradually enhances the differences in lateral positions (see Supplementary Movie S1). We choose an expansion region, 4.25-cm downstream of the inlet (Fig. 4a) for the measurement (channel width is around 335 µm). Similar to the straight rectangular microchannel region, the distance to the channel centerline decreases with increasing AR at the expansion region (Fig. 4c and d). The differences in lateral equilibrium positions for *E. gracilis* cells with different ARs at the expansion region is greater than the differences in the straight region (Fig. 3). Relative differences in averaged lateral positions between *E. gracilis* cells with AR = 1 and others (i.e. AR = 2–7) at straight region and expansion region are compared and presented in Supplementary Fig. S2. For example, at Re = 77 the averaged lateral position difference between *E. gracilis* cells with AR = 1 and AR = 7 is 45.1 µm at the expansion region, which is 8.5 times as large as that at the straight channel region (i.e. 5.3 µm). This expansion in distance is expected given the corresponding spreading of the focusing streamlines by 8.375-fold due to channel width expansion. This observation agrees with the previous experimental study by Hur *et al*.^28^, in which an expansion region was used to separate and enrich mammalian cells based on deformability.

### Separation and enrichment of *E. gracilis* by shape at the outlets

**Figure 5:**
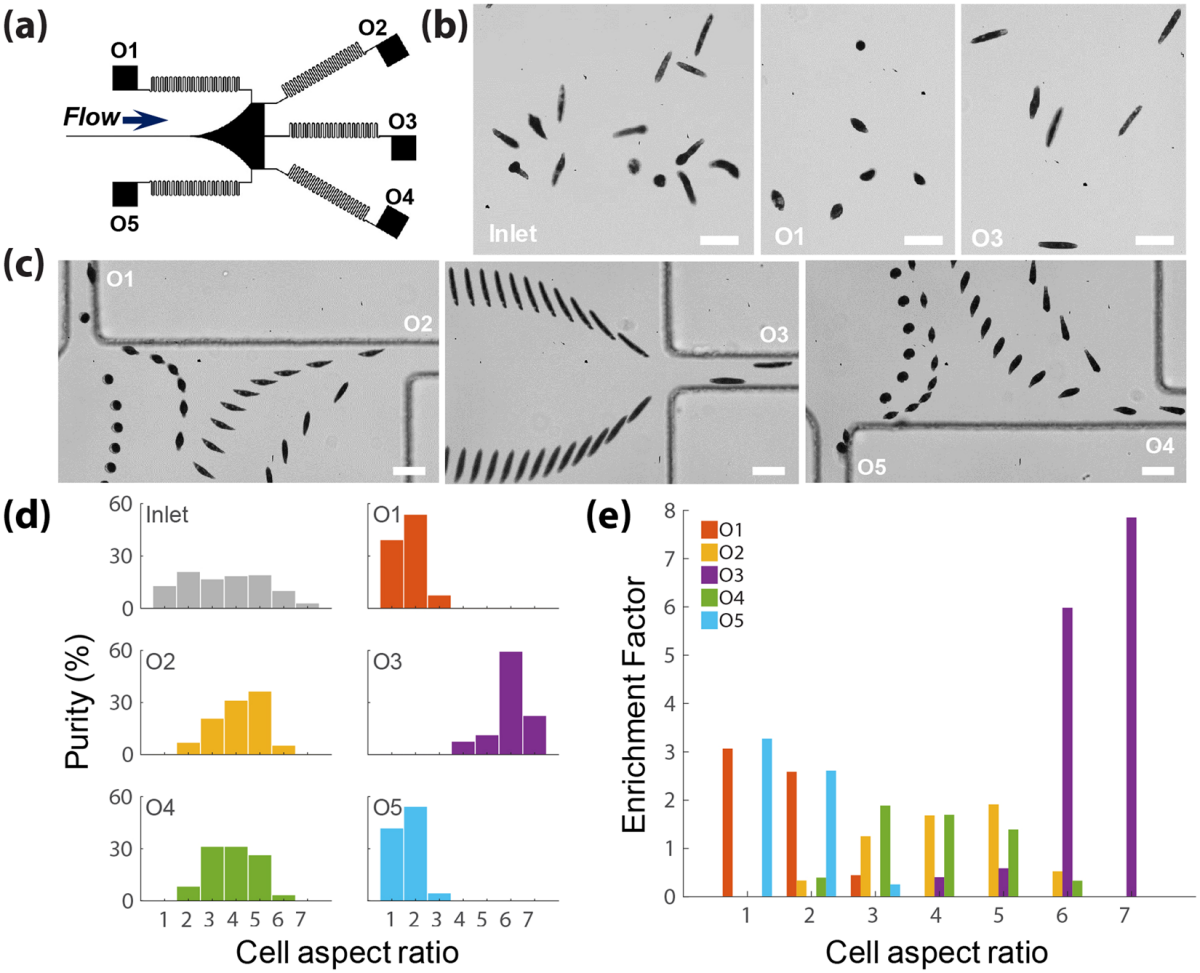
Shape-based separation of *E. gracilis* cells with different ARs ranging from 1 to 7 at the outlets at Re = 77. (**a**) An illustration of five outlets with high fluidic resistance (i.e. serpentine channels). (**b**) Snap-shot pictures comparing *E. gracilis* proportion at the inlet and outlets. (**c**) Superimposed experimental images showing that *E. gracilis* with different ARs are more likely to exit from different outlets: spherical shaped and long rod-shaped cells exit from outlets closer to the sidewall and centerline, respectively. Scale bar = 40 µm (**d**) A comparison of purity for *E. gracilis* cells with different ARs ranging from 1 to 7 at inlet and each outlet. (**e**) Bar graphs of enrichment factors for *E. gracilis* cells with different ARs for each outlet. At least 100 cells were measured for inlet and each outlet.

By taking advantages of differences in AR-dependent lateral equilibrium positions demonstrated above, we have performed shape-based separation and enrichment of *E. gracilis* cells with different ARs ranging from 1 to 7. Collection of cells with different ARs at different outlets with a flow rate of 300 µL/min (Re = 77) is shown (Fig. 5a–c). Each outlet has a fluidic resistor (i.e. a serpentine microchannel) with high resistance, which is designed to minimize the flow ratio distortion caused by any small variation (i.e. tubing length and debris clogging) in the outlet fluidic resistance^28^. *E. gracilis* cells with lower ARs (i.e. 1 and 2) are found to mostly exit from outlets 1 and 5 (see also Supplementary Movie S2), while the cells with higher AR (i.e. 6 and 7) are mainly captured from outlet 3 (see also Supplementary Movie S3). We use two parameters to quantify the separation efficiency: purity represents the proportion of cells with a specific AR at the inlet and each outlet; enrichment factor is defined as the proportion of cells with a specific AR in a given outlet to its proportion at the inlet. We have collected lower AR cells (AR = 1 and 2) at outlet 1 and outlet 5 with a purity of 92.6% and 96.8%, respectively; and higher AR cells (AR = 6 and 7) at outlet 3 with purity of 82.4% (Fig. 5d). Moreover, the enrichment factors for *E. gracilis* cells with AR = 1 at outlets 1 are 3.1 and 3.3, respectively, and the enrichment factors for cells with AR = 6 and 7 at outlet 3 are 5.9 and 7.8, respectively (Fig. 5e). *E. gracilis* cells with a middle range ARs (i.e. 3–5) are more likely to exit from outlets 2 and 4, with up to 87% purity and around 1.5 enrichment factor.

## Discussion

Cell viability tests were carried out using FDA and PI, which show that *E. gracilis* cells remain highly viable after flowing through the microchannel under inertial effects (see Supplementary Fig. S3). Moreover, since the rapid processing of the device does not lead to significant effect on gene expression over the time course of a separation^28^, the proposed platform is desirable for various downstream applications where transcriptomic analyses are required. We utilized dilute cell suspension (i.e. 10^5^ cells/mL) in the experiment in order to avoid particle-particle interactions, leading to the defocusing of cells at the expanding region, which adversely affect cell separation at the outlets^32^. It has been shown that particle concentration is an important factor affecting focusing behavior and accuracy. Inertially influenced particle-particle hydrodynamic interactions lead to multiple particle strains in the lateral direction as concentration is increased^33^. Moreover, the broadening of focused particle streams at expanding regions appeared at high particle suspensions^32^. At the expanding region, particles slow down and move closer due to expanding streams, which lead to an increase in particle–particle hydrodynamic interactions.

The throughput of the device can reach 800 µL/min (~1,300 cells/sec), which could be improved by optimizing cell concentration and Re, and using the design in a parallel network. Note that fluidic resistors located in the respective outlets could be tuned to capture enriched fractions of cells having particular particle shapes by adjusting the amount of fluid that splits into each outlet^28^. Further improvement and optimization of the channel parameters, i.e. number and lateral positions of outlets, flow rates (or Re) are also expected to increase enrichment factors for *E. gracilis* with specific AR(s). Moreover, the purity and enhancement factor could be increased by using the devices in a cascade configuration. Therefore, we are able to obtain high-purity *E. gracilis* cell samples with a specific AR (or shape) according to the specific application requirements.

In this work, we demonstrate passive, label-free, and high-throughput separation of *E. gracilis* microalgae by shape using inertial microfluidics. Under the combined effects of two inertial lift forces, a shear-gradient lift force and a wall-effect lift force, *E. gracilis* cells with various ARs migrate to different AR-dependent lateral dynamic equilibrium positions when flowing through a high aspect ratio rectangular microchannel. The distance to the channel centerline is shown to decrease with increasing cellular AR. The expansion region downstream of the straight microchannel enhances the differences in equilibrium positions, while maintaining cells in the focusing streamlines. Effective shape-based separation and enrichment of *E. gracilis* cells are achieved at the outlets: cells with lower ARs (i.e. 1 and 2), higher ARs (i.e. 6 and 7), and middle range ARs (i.e. 3–5) are found to mostly exit from the outlets closest to the channel walls, the outlet in the channel center, and the ones between the center and the sidewalls, respectively. Since the cell AR (or shape) of *E. gracilis* is an important biological, metabolic, and environmental marker, this capability enables a large range of industrial, research and clinical applications. Moreover, by integrating with metabolic and genetic engineering techniques, it is envisioned that this platform can be used as a powerful tool to develop enhanced *E. gracilis* cells with desirable properties^34–37^.

## Methods

### Microfluidic device design and fabrication

The microfluidic channel was fabricated with polydimethylsiloxane (PDMS) using standard soft-lithography techniques^38^. The inlet enables a relatively smooth pressure transition which reduces recirculations, where optional filters could also be placed to prevent the channel from be clogged by aggregates. The inertial focusing channel was designed to ensure that cells reach two lateral equilibrium positions along the longer faces of rectangular channel at the end. The basic parameter design rules, i.e. channel length required for focusing, flow rate, and channel cross-section can be found in our previous review articles^26, 27^. The whole microchannel has a uniform height of 90 µm. The width and length of the straight channel is 40 µm and 4.2 cm, respectively. It is preferable to have a smooth transition from the inertial focusing microchannel to the downstream expanding region that first gradually increases to avoid the formation of vortices that can trap and disturb cell trajectories, but then rapidly extends the cross-sectional area of the expanding region in a short distance as one moves along the direction of the flow, to sufficiently expand the distance between differentially-focused cells. In this regard, the expansion region was designed by increasing the angle between the channel wall and flow direction by 2° per 100 µm^28^. The serpentine microchannels serving as fluidic resistors at the outlets is composed of 20 U-shaped periods with a channel width of 40 μm and a total path length of around 2.5 cm.

### Cell culture

*Euglena gracilis Z* (NIES-48) was obtained from Microbial Culture Collection at National Institute for Environmental Studies (NIES), Japan. *E. gracilis* cells were cultured autotrophically in 50 ml tubes using Cramer-Myers (CM) medium^39^ at pH = 5.5. The cultures were exposed to a continuous light with an intensity of 100 μmol photons m^−2^ s^−1^, aerated with filter-sterilized mixture of carbon dioxide and air in a volumetric ratio of 15% at a flow rate of 1 L/min, and incubated at a constant temperature of 29 °C. Due to biological, metabolic, and environmental differences, the cultured *E. gracilis* cells have different shapes and sizes. Viability of *E. gracilis* cells was examined using fluorescence-based live-dead assays with fluorescein diacetate (FDA, Sigma-Aldrich) and propidium iodide (PI, Sigma-Aldrich), which can stain viable and dead cells, respectively (see Supplementary Fig. S4). The concentrations of FDA and PI are 10 µg/mL and 20 µg/mL, respectively, in phosphate buffered saline (PBS, Thermo Fisher Scientific).

### High-speed imaging and data analysis

*E gracilis* suspensions with a concentration of around 10^5^ cells/mL were injected into the microchannel at flow rates ranging from 300 to 800 µL/min using a syringe pump (Harvard Apparatus, MA, USA). The motion and behaviors of *E. gracilis* cells were monitored and recorded using an inverted microscope (Nikon Ti, Japan) equipped with a high-speed camera (Vision Research, NJ, USA). The high-speed images were captured with an exposure time of 1 µs, and frame rates were varied according to the channel region for measurement. Recorded videos and image sequences were processed using Phantom Camera Control Software (PCC) (https://www.phantomhighspeed.com/) and a custom-built MATALB routine (MathWorks, MA, USA). The code determines the center, maximum/minimum length (or AR) of individual *E. gracilis* cells in each image frame. The cell lateral position is calculated by measuring the distance between the cell center and the channel centerline.

## Electronic supplementary material

Supplementary Movie S1

Supplementary Movie S2

Supplementary Movie S3

Supplementary Information
