# Supplementary material for: Shape-based separation of microalga Euglena gracilis using inertial microfluidics
Source: Sci Rep. 2017 Sep 7;7:10802. doi: 10.1038/s41598-017-10452-5 (PMC5589772; doi:10.1038/s41598-017-10452-5)
Supplement: Supplementary file 4 — Supplementary Information [file 41598_2017_10452_MOESM4_ESM.pdf]

Supplementary Information

Shape-based separation of microalga *Euglena gracilis* using inertial microfluidics

Ming Li[1,2], Hector Enrique Muñoz[1], Keisuke Goda[2,3,4*], and Dino Di Carlo[1,5,6*]
[1]Department of Bioengineering, University of California, Los Angeles, USA
[2]Department of Electrical Engineering, University of California, Los Angeles, USA
[3]Department of Chemistry, University of Tokyo, Japan
[4]Japan Science and Technology Agency, Japan
[5]California NanoSystems Institute, University of California, Los Angeles, USA
[6]Jonsson Comprehensive Cancer Centre, University of California, Los Angeles, USA
[*] dicarlo@seas.ucla.edu, goda@chem.s.u-tokyo.ac.jp

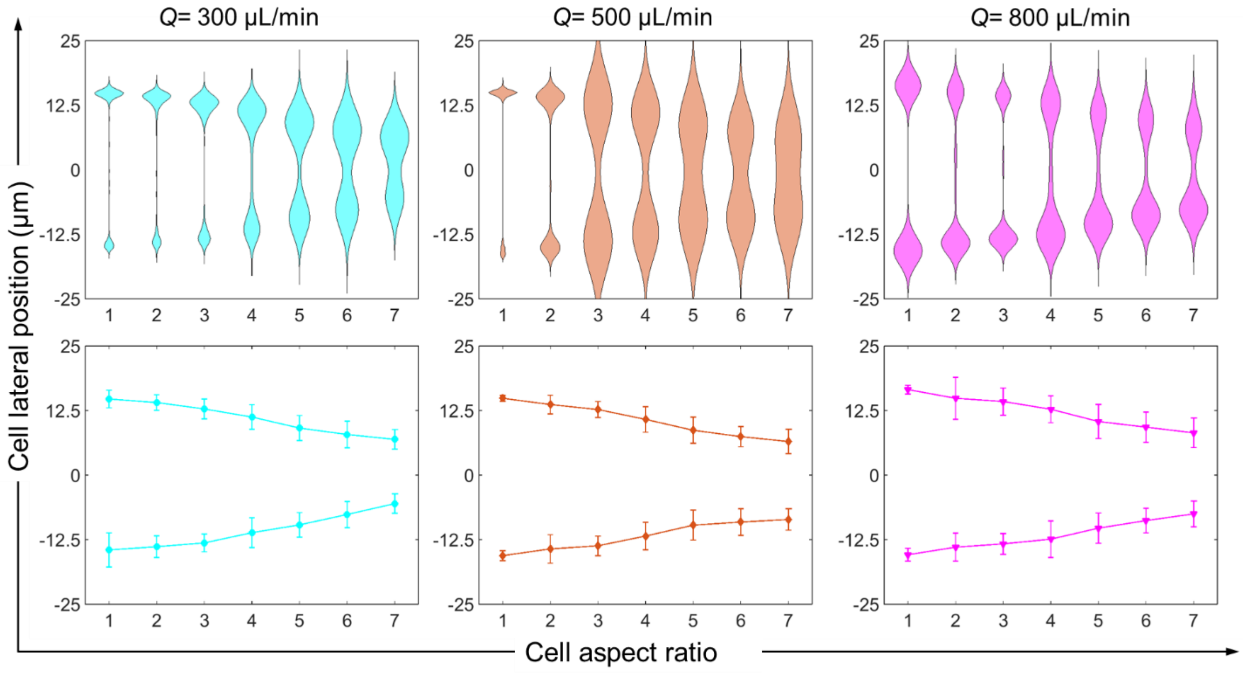

Figure S1 Inertial focusing distributions of *E. gracilis* cells with various aspect ratios ranging from 1 to 7 in a microchannel (width=50 μm, height=90 μm) at flow rates of 300, 500, and 800 μL/min.

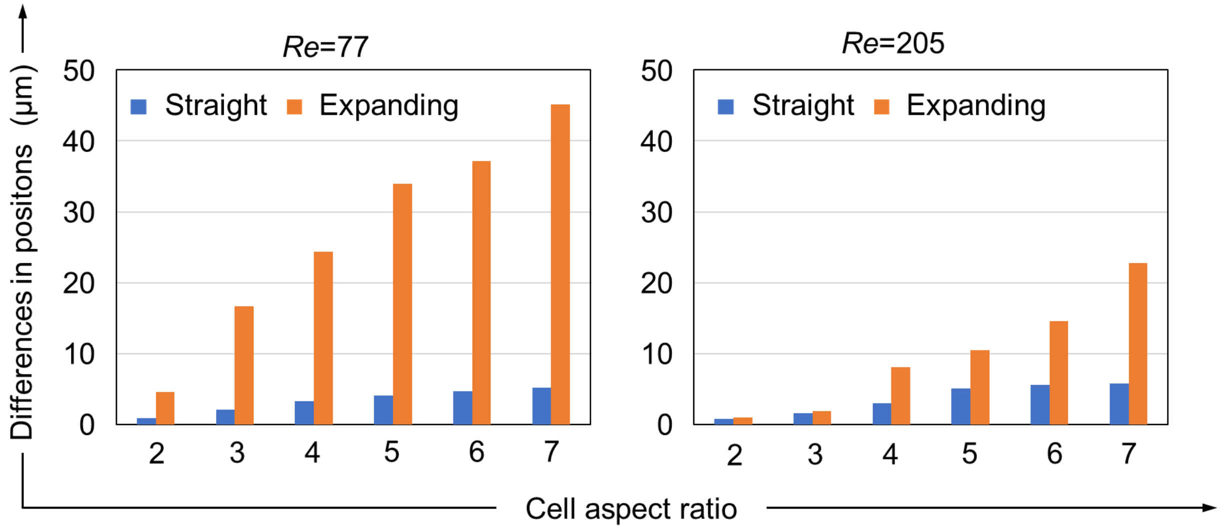

Figure S2 Comparison of differences in averaged lateral positions between *E. gracilis* cells with AR=1 and others (i.e. AR=2-7) in the straight region and the gradually expanding region for Re= 77 (left) and *Re*=205 (right).

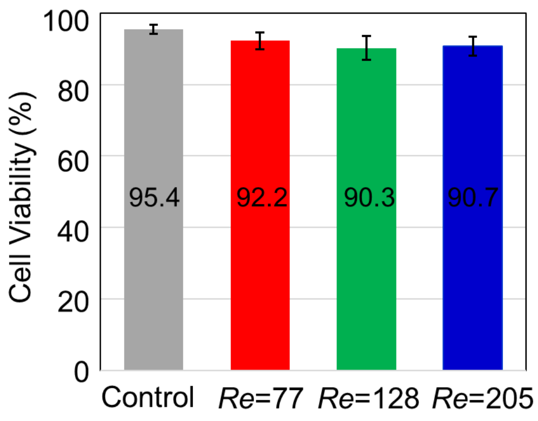

Figure S3 The viability of *E. gracilis* cells was not significantly affected by inertial effects. The standard deviations are obtained from three measurement.

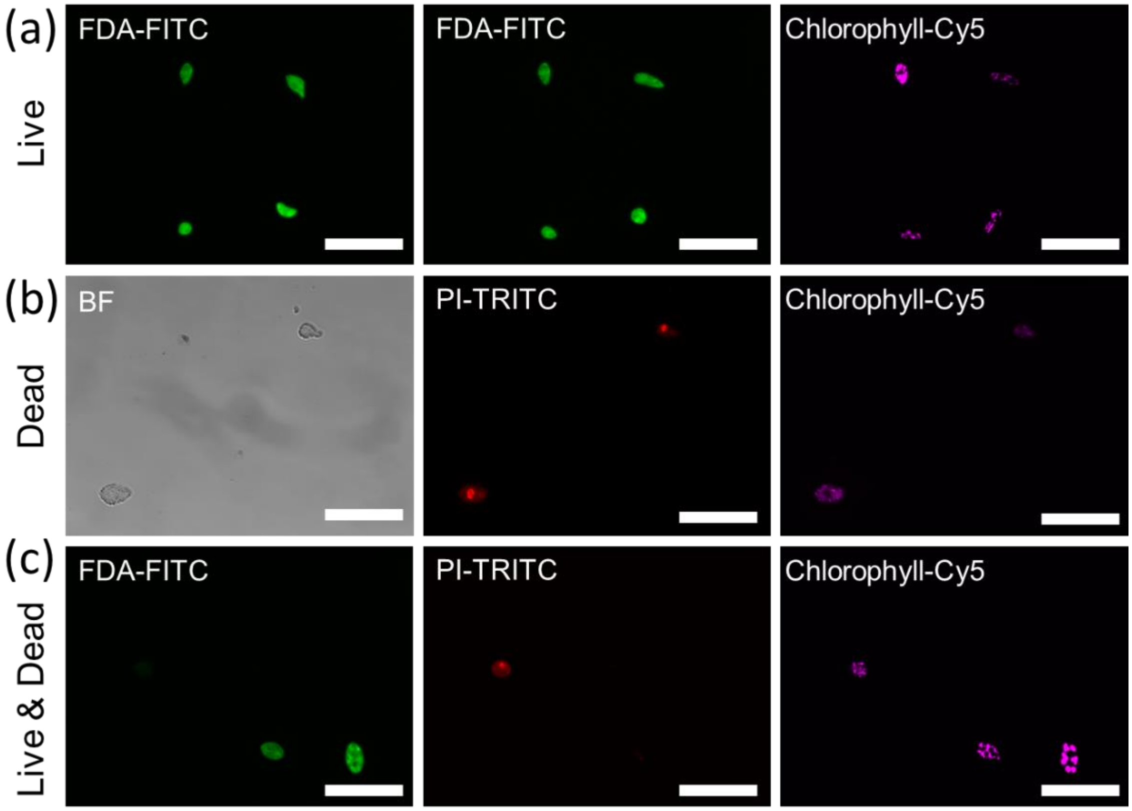

Figure S4 Viability assays of *E. gracilis* cells using fluorescence-based live-dead assays with FDA and PI. (a) Microscopic images of dead cells (from left to right: FDA signal in FITC channel, FDA signal in FITC channel, and chlorophyll auto-fluorescence signal in Cy5 channel). (b) Microscopic images of live cells (from left to right: bright field, PI signal in TRITC channel, and chlorophyll auto-fluorescence signal in Cy5 channel). (c) Microscopic images of live/dead cell mixtures (from left to right: FDA signal in FITC channel, PI signal in TRITC channel, and chlorophyll auto-fluorescence signal in Cy5 channel). Scale bar=50 µm.

Supplementary Movie Captions

Movie S1

Shape-dependent lateral inertial focusing equilibrium positions for *E. gracilis* cells at an expansion region. High-speed microscopic video at a flow rate of 300 µL/min (Re= 77).

Movie S2

Shape based separation of *E. gracilis* cells at outlets 1 and 2. High-speed microscopic video at a flow rate of 300 µL/min (Re= 77).

Movie S3

Shape based separation of *E. gracilis* cells at outlet 3. High-speed microscopic video at a flow rate of 300 µL/min (Re= 77).
